# Machine learning is the key to diagnose COVID-19: a proof-of-concept study

**DOI:** 10.1038/s41598-021-86735-9

**Published:** 2021-03-30

**Authors:** Cedric Gangloff, Sonia Rafi, Guillaume Bouzillé, Louis Soulat, Marc Cuggia

**Affiliations:** 1 Univ Rennes, CHU Rennes, INSERM, LTSI-UMR 1099, F-35000 Rennes, France; 2 Department of Emergency Medicine, CHU Rennes, F-35000 Rennes, France

**Keywords:** Infectious diseases, Hypoxia

## Abstract

The reverse transcription-polymerase chain reaction (RT-PCR) assay is the accepted standard for coronavirus disease 2019 (COVID-19) diagnosis. As any test, RT-PCR provides false negative results that can be rectified by clinicians by confronting clinical, biological and imaging data. The combination of RT-PCR and chest-CT could improve diagnosis performance, but this would requires considerable resources for its rapid use in all patients with suspected COVID-19. The potential contribution of machine learning in this situation has not been fully evaluated. The objective of this study was to develop and evaluate machine learning models using routine clinical and laboratory data to improve the performance of RT-PCR and chest-CT for COVID-19 diagnosis among post-emergency hospitalized patients. All adults admitted to the ED for suspected COVID-19, and then hospitalized at Rennes academic hospital, France, between March 20, 2020 and May 5, 2020 were included in the study. Three model types were created: logistic regression, random forest, and neural network. Each model was trained to diagnose COVID-19 using different sets of variables. Area under the receiving operator characteristics curve (AUC) was the primary outcome to evaluate model’s performances. 536 patients were included in the study: 106 in the COVID group, 430 in the NOT-COVID group. The AUC values of chest-CT and RT-PCR increased from 0.778 to 0.892 and from 0.852 to 0.930, respectively, with the contribution of machine learning. After generalization, machine learning models will allow increasing chest-CT and RT-PCR performances for COVID-19 diagnosis.

## Introduction

The severe acute respiratory syndrome coronavirus 2 (SARS-coV-2) outbreak started in December 2019 in the Hubei province, China. The associated disease, coronavirus disease 2019 (COVID-19)^1^, has now spread worldwide. The World Health Organization currently reports more than 10 million confirmed cases and 500,000 deaths. Increased mortality rates and the collapse of healthcare systems have been reported in several regions^2–4^. Indeed, due to SARS-coV-2 contagiousness, promiscuity within health systems can promote patient-to-patient transmission^5,6^ and the contamination of healthcare workers^7^, rapidly leading to the saturation of health systems^8^. To limit this effect, patients with COVID-19 infection are hospitalized in specific units after being emergency department (ED) triage^9^. Therefore, it is essential to have a reliable and easy-to-use tool for COVID-19 diagnosis. SARS-coV-2 real-time RT-PCR reverse transcription–polymerase chain reaction (RT-PCR) is the accepted standard for COVID-19 diagnosis^10^. However, RT-PCR performances are sub-optimal and, like for any other test, there are false negatives results^11,12^. Therefore, additional investigations should be performed in patients with negative RT-PCR results but high clinical probability of COVID-19. In this context, chest-CT is an interesting tool because it allows detecting virus-induced lung tissue damages and alternative diagnoses^13^. Thus, when a patient presents a high clinical probability of COVID-19, a negative RT-PCR and a chest-CT showing typical COVID-19 lesions with no sign of alternative diagnosis, it is possible to consider that the patient has COVID-19 with a false negative RT-PCR result. The use of chest-CT alone cannot be recommended, but its combined use with clinic and RT-PCR allows to resolve diagnostic ambiguities^14^. However, RT-PCR and chest-CT cannot be performed in all patients suspected to have COVID-19 for many reasons, including reagent shortage^15^, device unavailability, lack of human resources, and high costs. Moreover, the time required to perform both tests increase the risk of ED overcrowding by patients waiting for their results. Therefore, health professionals must adapt their diagnostic strategies in function of their resources^16^. To our knowledge, the potential contribution of machine learning using imaging, clinical and laboratory data has been poorly evaluated in this context. Machine learning is an inherited artificial intelligence approach that enables computers to extract or classify patterns. It allows predicting whether a patient belongs to a predefined group using explanatory variables. The recent increase in machine learning models in the healthcare field suggests that these methods could improve the COVID-19 diagnostic strategy^17^. The objective of this study was to develop and evaluate machine learning models using clinico-biological data from health records to improve the RT-PCR and chest-CT performances for COVID-19 diagnosis among post-emergency hospitalized patients.

## Materials and methods

This study protocol was approved by the Medical Ethics Committee of Rennes academic hospital (approval number 0020.93 issued on July 7, 2020). All methods were performed in accordance with the relevant guidelines and regulations. Authorization to conduct research from the Clinical Data Warehouse of Hospital of Rennes was given by CNIL—Commission Nationale Informatique et Liberté (Authorization number 2020-028 issued on February 27, 2020). Informed written consent from each participant was not required for this study according to the French Data Protection Act of 6 January 1978, as this study only included information from existing medical records and did not involve interaction with patients or collection of identifiable private information. Each entry of sample data was deidentified to ensure confidentiality.

### Software

Data extractions, manipulations, statistical analyses, and model buildings were performed with “R-studio”, version 1.3.1093, RStudio PBC, 2009–2020. Specialized packages and functions were used for specific analysis: “Dplyr”, version 1.0.0 was used for data manipulation, “Purrr”, version 0.3.4 for data simplification, and “missForest”, version 1.4 for missing data imputation. Variable importance calculations and K-fold cross-validation were performed with the “Caret” package, version 6.0-86. Correlations matrix were calculated with the “corrplot” package, version 0.84. Random forests were built with “randomForest” version 4.6-14 and artificial neural networks with “neuralnet” version 1.44.2. “pROC” version 1.16.2 was used to generate the receiver operating characteristic (ROC) curves and calculate the area under the curve (AUC) for each model.

### Setting

Data were collected retrospectively from patients admitted to the adult E.D. of Rennes Academic Hospital, France.

### Patient selection

All post-emergency hospitalized patients ≥ 18 years old admitted between March 20, 2020 and May 5, 2020 and suspected to have COVID-19 were included in the study.

### Data collection

Data were automatically collected from “eHOP”, a local clinical data warehouse in which health data are integrated and de-identified in real time^18^. Structured data, such as laboratory results, were directly collected from the data warehouse. Text fields were structured by using regular expressions^19^.

### Data pre-processing

**Figure 1:**
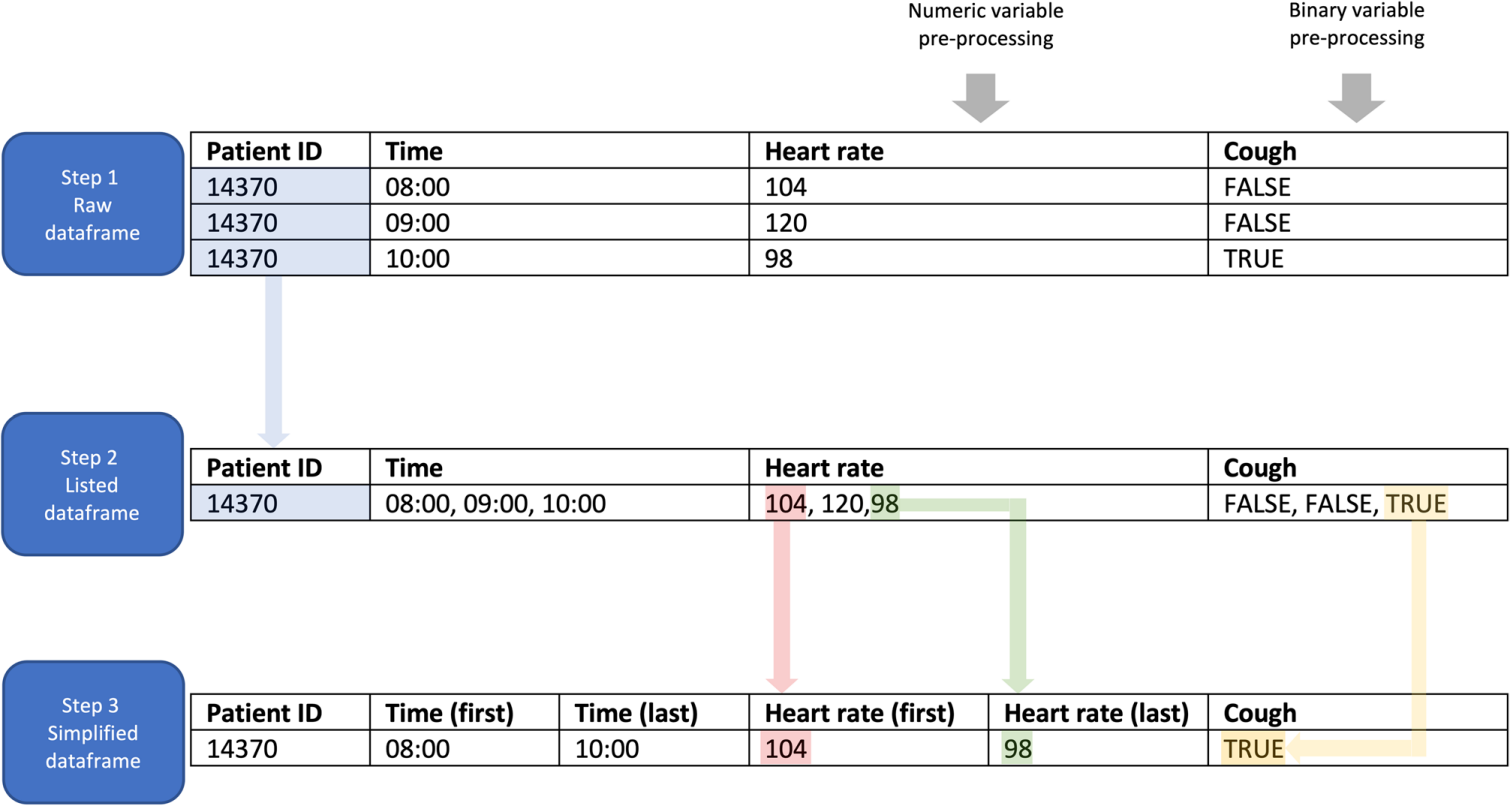
Data pre-processing. *The first step* corresponded to raw data, as they were initially stored in the database. Each ID was characterized by multiple rows. *On the second step*, data were listed in chronological order, with a single row per ID (blue arrow). *In the third step*, data were simplified. For numeric variables, only the first value was selected (red arrow). For binary variables, the value “true” was retained when it was present at least once in the list (yellow arrow).

In the raw data-frame, all values were associated with a unique identifier (ID) corresponding to each patient’s admission. This data-frame contained multiple lines per ID (Fig. 1, step 1). Variables collected more than once during the patient journey appeared as lists (Fig. 1, step 2). Lists were simplified according to the type of variable (Fig. 1, step 3).

### Predicted variable

The predicted variable for each patient was the presence of COVID-19. COVID-19 was diagnosed as follows. Patients were triaged at ED admission and considered “suspected COVID-19” when they had at least one symptom compatible with COVID-19. Symptoms considered as compatible with COVID-19 were the followings: cough, dyspnea, hyperthermia, myalgias, asthenia, diarrhea, confusion or anosmia. After triage, patients were examined by an ED physician who estimated the clinical probability of COVID-19 (low, intermediate or high) and the need for hospitalization. RT-PCR and chest-CT were performed in all hospitalized patient suspected to have COVID-19. When suspected COVID-19 patients had positive RT-PCR, they were considered “COVID-19 positive”, regardless of the level of clinical probability. When clinical probability was “high” and chest-CT showed typical COVID-19 images with no sign of alternate diagnosis, the patient was considered “COVID-19 positive”, even if RT-PCR was negative. In this case, the RT-PCR result was considered a false negative^20,21^. Patients were allocated to “COVID” and “NOT-COVID” groups accordingly.

### Predicting variables selection

All clinical and laboratory variables present in the database were collected. The Student’s *t*- and chi-square tests were used to compare means between groups for numerical and binary variables, respectively. A p value < 0.05 was considered statistically significant. Variables with a p value < 0.2 were considered variables of interest. To avoid multicollinearity, correlation coefficients were calculated for each pair of variables of interest. When correlation coefficient was higher than 0.8, one of the two variables was excluded.

### Data split

Data were randomly divided in two parts: the train data-frame, and the test data- frame. The train data-frame corresponded to 80% of the whole data-frame and was used to build the models. Models performances were evaluated using the test data-frame that corresponded to the remaining 20%.

### Missing data imputation

Before the training process, missing values were imputed independently for each data-frame with a non-parametric procedure developed by Stekhoven and Buhlmann. This method called “missForest” is well-suited for mixed datasets requiring categorical and continuous variables imputations and is based on a random forest model trained iteratively. In this method, an evaluation is made after each iteration by calculation of the normalized root mean squared error and implementation is stopped when the evaluation indicates a decrease in performance. Three iteration were performed with 100 tree per random forest in this study.

### Model training

Three model types were constructed: binary logistic regressions, random forests and artificial neural networks. Random forest models were trained with 500 trees; neural networks were composed of three layers. Each model type was trained with three sets of variables: clinico-biological variables, clinico-biological variables with chest-CT, and clinico-biological variables with RT-PCR. A k-fold cross validation was performed in order to prevent over-fitting. Overfitting occurs when à machine learning algorithm captures the noise of the data. In this case, high performances are observed on the training data, but poor results are observed on new data. In other words, overfitted models cannot give suitable predictions on new patients. K-folds cross validation is a high-performance method to prevent overfitting. In this approach, the data-frame is divided into k parts called “folds”. A model is trained by using k − 1 folds, and the remaining fold is used to validate the model. The same procedure is applied k times (once per fold). This approach is well-suited for small datasets, but requires more calculations. In this study, k = 10 folds were used to build the models.

### Variable importance

To compare the importance of the different variables, the value of the most important variable in each model was arbitrarily set at 100 and the relative importance of each variable was determined with an adequate method depending on the model. In binary logistic regressions, the absolute value of the t-statistic for each parameter was used to calculate the importance of each variable. In random forests, the prediction error on the out-of-bag portion of the data was recorded for each tree and he same was done after permuting each predictor variable. The difference between the two were averaged over all trees and normalized by the standard deviation of the differences to determine each variables importance. In the neural network models, the method was based on combinations of the absolute values of the weights^22^.

### Performance measurement

Models were built with the train data-frame and their performances were assessed on the test data-frame, whose data were not used for model-building. This procedure guarantees non-biased performances measurements by confronting the models to unseen data, as if they were challenged to predict the presence of COVID-19 among new patients. The area under ROC curves is commonly used to evaluate and compare classifiers in machine learning, biomedical and bioinformatics applications^23^. In this study, models’ predictions were compared to the “COVID” variable in the test data-frame and ROC curves were constructed accordingly. The AUC was the primary outcome used to evaluate each model performance.

### Ethics approval

This study was approved by the ethic committee of Rennes academic hospital (number of approval: 20.93).

### Consent for publication

All methods were performed in accordance with the relevant guidelines and regulations. Authorization to conduct research from the Clinical Data Warehouse of Hospital of Rennes was given by CNIL—Commission Nationale Informatique et Liberté (Authorization number 2020-028 issued on February 27, 2020). Informed written consent from each participant was not required for this study according to the French Data Protection Act of 6 January 1978, as this study only included information from existing medical records and did not involve interaction with patients or collection of identifiable private information. Each entry of sample data was deidentified to ensure confidentiality.

## Results

### Patient selection

**Figure 2:**
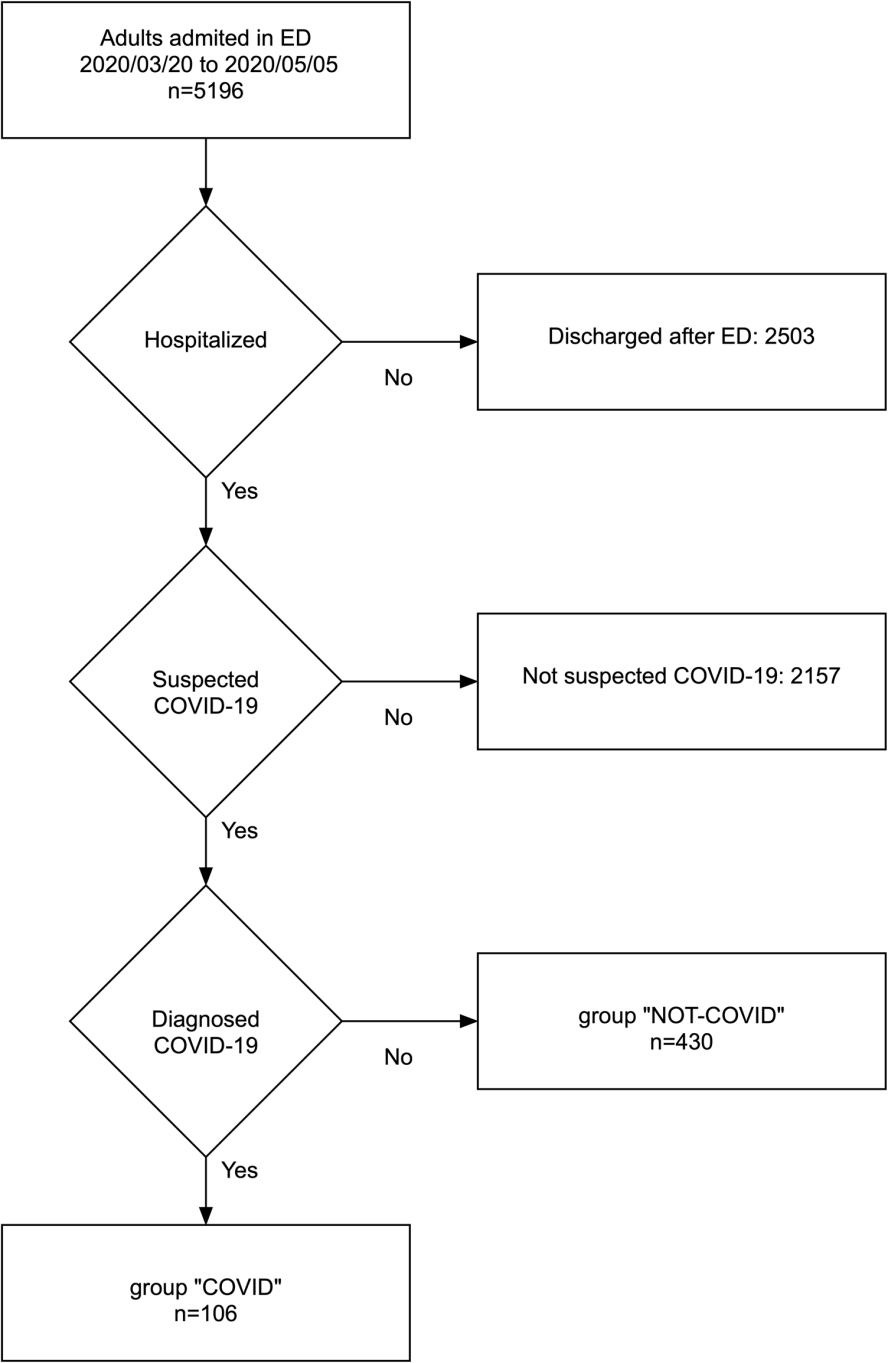
Flow chart of patient selection. Patients suspected to have COVID-19 had at least one of the following symptoms: cough, dyspnea, hyperthermia, myalgias, asthenia, diarrhea, confusion or anosmia. Both chest-CT and RT-PCR were performed in all patients with suspected COVID-19 who were hospitalized.

The patient selection flow chart is presented Fig. 2.

### Diagnostics

Diagnostics for the 536 patients selected in this study are represented in Table 1.

**Table 1:** Diagnostic categories for the 536 suspected COVID-19 hospitalized patients.

Diagnostic	n (%)
COVID-19	106 (19.8)
Cardiac insufficiency	98 (18.3)
Pneumonia	74 (13.8)
Chronic obstructive pulmonary disease (COPD)	52 (9.7)
Influenza-like illness	38 (7.1)
Intra-abdominal infection	34 (6.3)
Asthma	20 (3.7)
Non organic dyspnea	19 (3.5)
Urinary tract infection	19 (3.5)
Confusion in the elderly (delirium)	14 (2.6)
Transcient fever	14 (2.6)
Cancer	12 (2.2)
Pulmonary embolism	12 (2.2)
Skin infection	6 (1.1)
Others	5 (0.9)
Central nervous system infection	4 (0.7)
Heart infection (pericarditis, myocarditis, endocarditis)	4 (0.7)
Prothesis-related infection	3 (0.6)
Traumatic dyspnea	2 (0.4)
Total	536 (100)

All patients presented at last one clinical sign compatible with COVID-19 and underwent chest-CT and RT-PCR. 106 were classified in the COVID group, 430 in the NOT-COVID group.

### Selected variables

Twenty-three clinico-biological variables were considered as variables of interest (Table 2). Variables not selected as variables of interests are presented in supplementary Table 1.

**Table 2:** Variables of interest.

	NOT-COVID (n = 430)	COVID (n = 106)	P value
**Clinicals and treatments**			
Cough, %	83.0 (79.1–87.0	92.4 (86.5–98.2)	0.0563
Hyperthermia, %	66.7 (61.8–71.7)	77.2 (67.9–86.4)	0.0940
Myalgias, %	17.1 (13.2–21.1)	34.1 (23.7–44.6)	0.0012*
Asthenia, %	30.9 (26.1–35.8)	45.5 (34.5–56.5)	0.0187*
Diarrhea, %	22.9 (18.5–27.3)	32.9 (22.5–43.2)	0.0867
Confusion, %	21.7 (17.4–26.1)	7.5 (1.7–13.4)	0.0063*
Furosemid (usual treatment), %	16.0 (12.2–19.9)	6.3 (0.9–11.7)	0.0401*
**Arterial blood gas**			
Base excess, mmol/L	3.0 (2.6–3.4)	2.7 (1.8–3.6)	0.0151*
Lactates, mmol/L	1.7 (1.5–1.9)	1.3 (1.1–1.5)	< 0.001*
**Complete blood count**			
Red blood cell count, Tera/L	4.2 (4.1–4.3)	4.5 (4.3–4.7)	< 0.001*
Mean platelet volume, fL	8.6 (8.4–8.8)	8.8 (8.5–9.1)	0.0269*
Leukocytes, G/L	10.2 (9.6–10.8)	7.7 (6.7–8.7)	0.0568
Neutrophils, G/L	7.9 (7.4–8.4)	6 (5.1–6.9)	0.1488
Platelet count	236.1 (225.8–246.4)	198.9 (182.1–215.7)	0.0482*
Eosinophils percentage	1.4 (1.1–1.7)	0.8 (0.4–1.2)	0.0873
Basophils percentage	0.6 (0.5–0.7)	0.4 (0.3–0.5)	< 0.001*
Lymphocytes, G/L	1.3 (1.2–1.4)	1 (0.8–1.2)	< 0.001*
Monocytes, G/L	0.8 (0.7–0.9)	0.6 (0.5–0.7)	< 0.001*
**Ionogram**			
Potassium, mmol/L	4.1 (4–4.2)	4 (3.8–4.2)	0.0039*
Phosphor, mmol/L	1 (0.9–1.1)	1.1 (0.9–1.3)	< 0.001*
**Hemostasis and liver enzymes**			
Alanine aminotransferase, mmol/L	64.5 (47.1–81.9)	46.2 (33.9–58.5)	0.1845
International normalized ratio	1.3 (1.2–1.4)	1.2 (1.1–1.3)	< 0.001*
d-Dimer, ng/ml	2200 (1600–2800)	2800 (1400–4200)	< 0.001*

Means and percentage between groups were compared with Student’s t- and chi-square tests, respectively. Only variables with p < 0.02 were considered as variables of interest and were listed in this table. Values in brackets represent the 95% confidence intervals. *p < 0.005.

### Variables correlations

**Figure 3:**
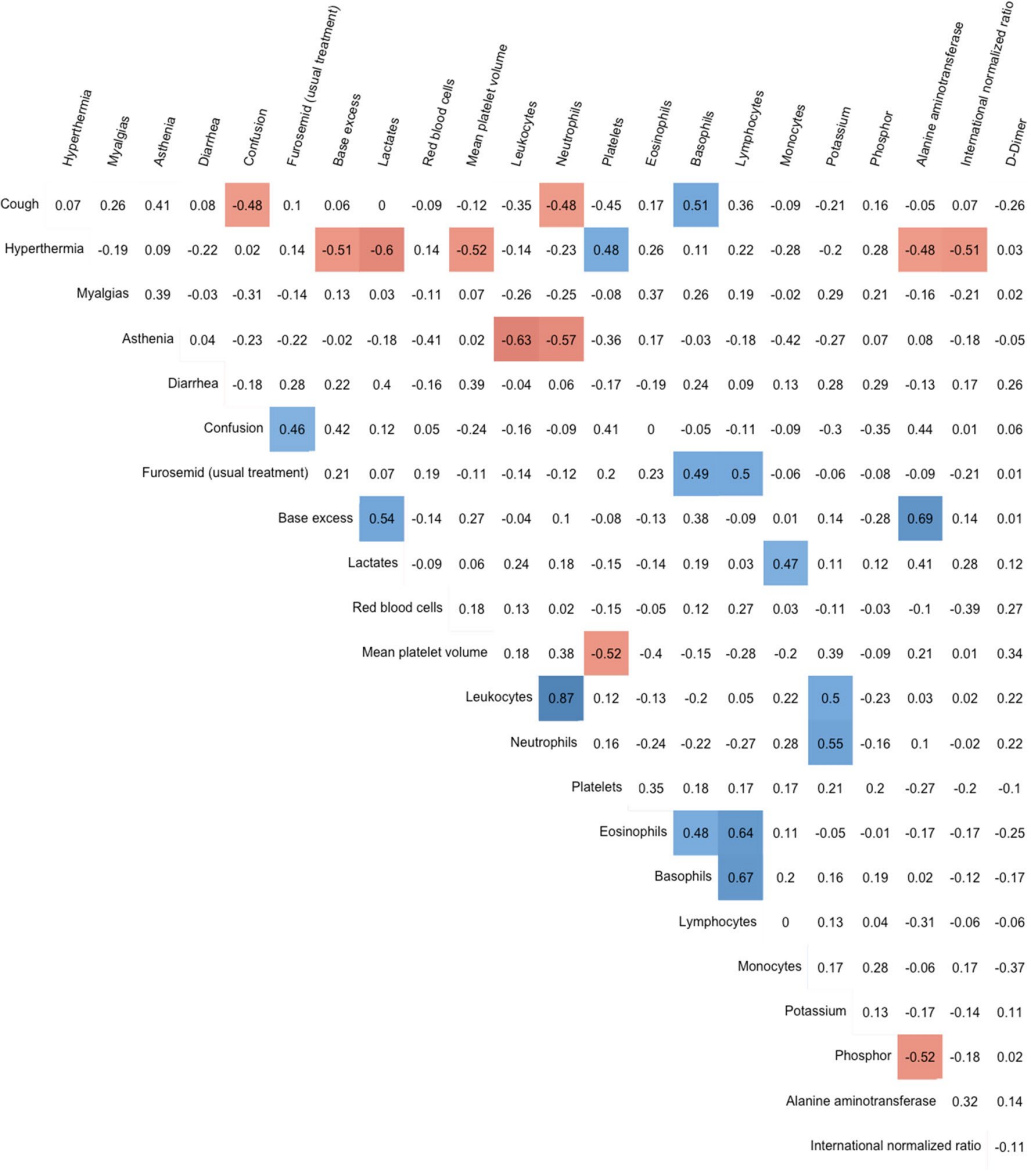
Correlation coefficients for all pairs of variables of interest. Pearson and Spearman coefficients were calculated for continuous and binary variables, respectively. Correlations not significantly different from 0 are in white cells. Positive correlations are in blue cells, and negative correlations in red cells. Leukocyte count and neutrophil count were identified as highly correlated, and leukocyte count was removed from model building.

Calculation of the correlation coefficients for each pair of the 23 variables of interest (Fig. 3) showed that two variables were highly correlated with a correlation coefficient > 0.8: neutrophil count and leukocyte count. Leukocyte count was removed from model building. Therefore, the final set of clinical and laboratory variables selected for model building included 22 variables.

### Chest-CT and RT-PCR performances

AUCs of chest-CT and RT-PCR used alone for COVID-19 diagnosis were 0.778 (CI 95% 0.682–0.873) and 0.852 (CI 95% 0.764–0.940), respectively.

### Models performance

The AUC values for the three model types trained with each set of variables are presented in Table 3.

**Table 3:** AUC for each machine learning model.

	Clinico-biological	Clinico-biological + chest-CT	Clinico-biological + RT-PCR
Binary logistic regression	0.772 (0.668–0.875)	0.886 (0.804–0.968)	0.930 (0.867–0.992)
Random forest	0.754 (0.638–0.871)	0.829 (0.724–0.935)	0.903 (0.816–0.989)
Artificial neural network	0.728 (0.617–0.840)	0.892 (0.811–0.973)	0.844 (0.731–0.957)

Three model types were constructed: binary logistic regression, random forest, and artificial neural network. Each model was trained with three sets of variables: clinico-biological, clinico-biological with chest-CT, and clinico-biological with RT-PCR. Models were built and assessed on two separate data-frames. Values in brackets represent the 95% confidence intervals.

**Figure 4:**
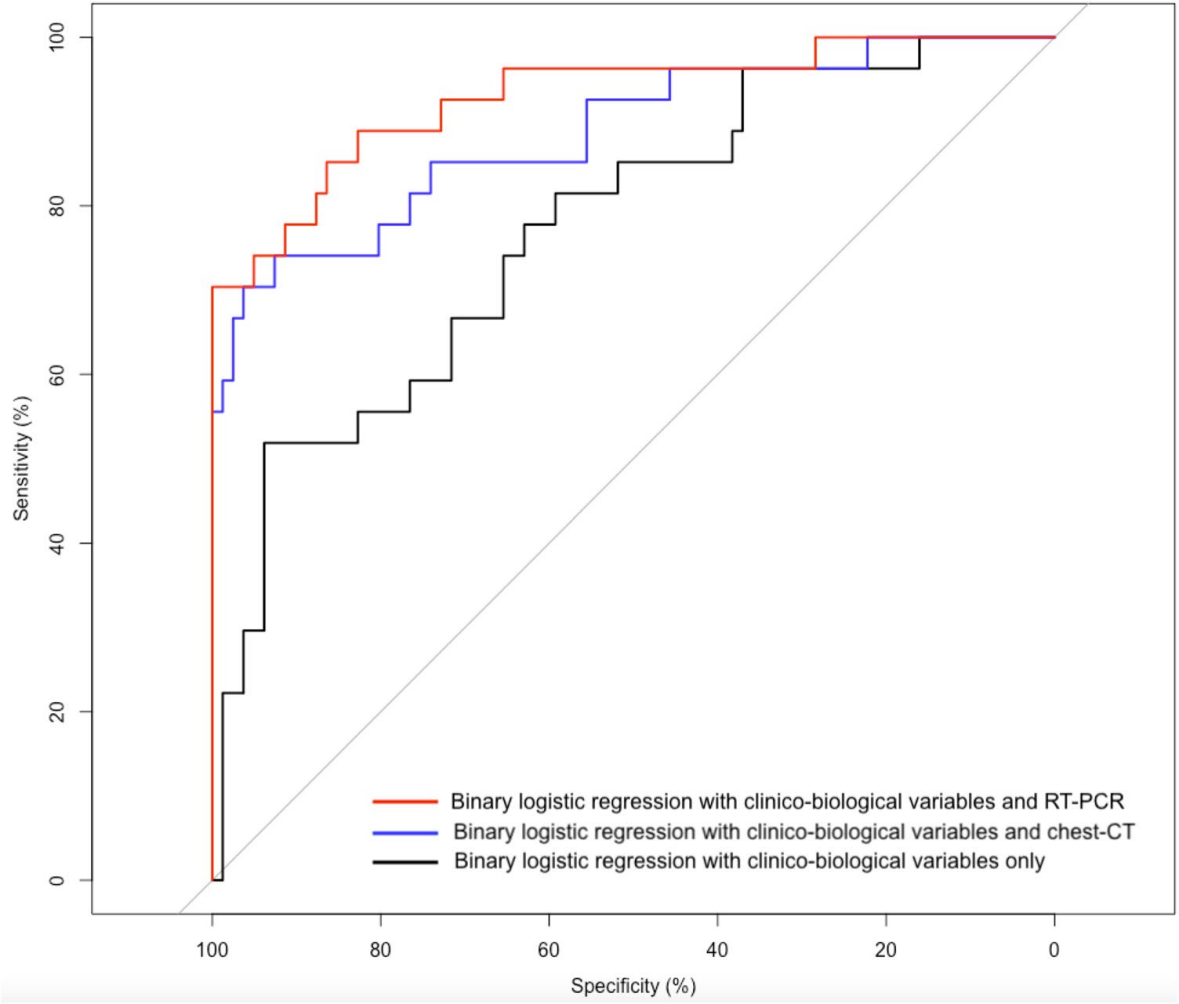
ROC curves for the 3 logistic regression models based on common clinico-biological variables alone, clinico-biological variables with chest-CT and common clinico-biological variables with RT-PCR. The “Binary logistic regression with clinico-biological variables and RT-PCR” was the best performing model in this study.

The ROC curves for the binary logistic regression models are presented Fig. 4.

### Importance of clinico-biological variables

The importance of the different variables in each model type is presented Table 4.

**Table 4:** Importance of clinico-biological variables by decreasing order in each model type.

	Binary logistic regression	Random forest	Artificial neural network
**Clinicals and treatments**			
Cough	62	1.5	21.3
Hyperthermia	24.7	3.1	24.1
Myalgias	40.5	9.36	39.7
Asthenia	24.1	6.7	34.1
Diarrhea	57.1	6	22.9
Confusion	91.8	5.8	33
Furosemid (usual treatment)	72.8	0	22.3
**Arterial blood gas**			
Base excess	31.6	37.3	16.2
Lactates	100	100	77.9
**Complete blood count**			
Red blood cell count	85.1	86.4	41
Mean platelet volume	0	34.5	0
Neutrophils	64.6	50.6	55.1
Platelet count	36.8	46.8	47.3
Eosinophils	35.9	36.5	57.1
Basophils	74.8	79.9	100
Lymphocytes	67.4	51.8	46
Monocytes	27.6	57.3	52.6
**Ionogram**			
Potassium	17.5	39.4	19.4
Phosphor	6.2	45.5	4.2
**Hemostasis and liver enzymes**			
Alanine aminotransferase	64.6	39.8	3.1
International normalized ratio	9	39.2	0.2
d-Dimer	57.6	58.4	10

The relative importance of each variable was calculated in comparison with the most important variable in the model, whose importance was arbitrarily set at 100.

## Discussion

### Models presented in this study were trained on typical suspected COVID-19 patients

All models were trained and evaluated using data from patients with diseases (e.g. heart failure, pneumonia, asthma, COPD; Table 1) that are frequently observed in ED and that share clinical symptoms with COVID-19. The finding that our machine learning models could differentiate between these diseases and COVID-19 suggests that they could be implemented in other EDs with similar patient populations.

### The variables selected for model-building were consistent with the clinico-biological signs of COVID-19

These variables belong to five categories: clinical signs, arterial blood gas, blood cell count, ionogram, hemostasis and liver enzymes. Clinical signs: the proportion of cough, hyperthermia, myalgia, asthenia, diarrhea, and confusion was significantly higher in the COVID-19 than in the NO-COVID-19 group. Such symptoms have previously been reported in numerous studies^24–28^. Interestingly, anosmia was not selected as a variable of interest, suggesting a lack of relevance of this symptom in our setting^29,30^. Arterial blood gas: in the NOT-COVID group, serum lactate concentration was higher, and base-excess was lower than in the COVID group, revealing the presence of patients with circulatory failure, a frequently reported complication of bacteremia^31^. Therefore, serum lactate concentration and base-excess are relevant for differentiating between patients with COVID-19 and with bacterial infections. Blood cell count: the mean leukocyte, lymphocyte, and platelet counts were lower in the COVID than in the NOT-COVID group. Previous authors have reported similar results. Indeed, a meta-analysis from Zhu et al*.* showed that patients with COVID-19 do not have hyperleukocytosis, except when associated with bacteremia^32^. COVID-19-associated lymphopenia correlates with the disease severity and is related to an immune response deficiency^33^. Similarly, thrombocytopenia was previously identified as a poor prognosis factor in this context^34^. Indeed, a meta- analysis by Lippi et al. revealed that platelet count was significantly lower in patients with severe COVID-19^35^, suggesting an inappropriate activation of the coagulation process. Ionogram: the mean potassium concentration was lower in the COVID group. This could be due to hyperventilation, but further investigation must be conducted to confirm this hypothesis. Hemostasis and liver enzymes: the mean d-dimer concentration was higher in the COVID group than in the NOT-COVID group. Elevated d-dimers are associated with higher rates of thromboembolic events^36^. These results are in line with the theory of an increased thromboembolic risk in patients with COVID-19^37–39^. This finding could be associated with the presence of antiphospholipid antibodies, but the pathophysiology of this phenomenon is still debated^40^. Variables selected for model building were therefore consistent with previous studies that have reported clinico-biological signs of COVID-19.

### Machine learning models will help to triage COVID-19 patients

RT-PCR and chest-CT are expensive, require qualified professionals to perform them and it is a real challenge to be able to get efficiently these two examinations in the context of a pandemic. An increasing number of patients awaiting results of these tests can lead to ED overcrowding and increased mortality rates in an epidemic context^41,42^. The logistic regression model presented in this study and trained only with clinico-biological variables had an AUC value of 0.754. This model only requires clinical examination and routine biology assays: complete blood cell count, ionogram, standard hemostasis tests, liver enzymes, and arterial blood gas. Such tests are low- cost and can be realized worldwide using automated devices. Therefore, in ED, a first triage identifying patients requiring isolation might be done by using machine learning while waiting for the RT-PCR result.

### Machine learning will improve RT-PCR and chest-CT performance for COVID-19 diagnosis

Several studies found sub-optimal performances of these tests when only one is used for COVID-19 diagnosis^11,13,43,44^. Indeed, the sensitivity of the RT-PCR test depends on the number of cycles used to determine the cut-off value for positivity^45^ and one of the issues by using chest-CT alone for COVID-19 diagnosis is the risk of false positive^20,46^. Artificial intelligence methods could be used to overcome these drawbacks. Some studies have already investigated the use of artificial intelligence for COVID-19 diagnosis and their number is progressively increasing with the pandemic duration^47,48^. Many of these studies are based on deep neural networks to improve COVID-19 diagnosis by chest-CT or X-ray imaging, particularly to help to differentiate between COVID-19 lesions and bacterial lung diseases^49–52^. For examples, the COVID-net tool based on 16,756 chest radiography images across 13,645 patients has an accuracy of 92.4%, the COVID-19 detection neural network (COVNet) based on 4356 chest-CT from 3322 patients has an accuracy of 95%^53,54^. However, few studies used laboratory, clinical, and imaging data together for COVID-19 diagnosis. Our results are in line with studies that used machine learning models based on clinico-biological variables for COVID-19 diagnosis^55–57^. The performances of these models were low, excepted for the model described by Plante and al. that used data from a large sample, but did not include imaging data^58^. Another study integrated RT-PCR, chest-CT and clinico-biological data, like in the present work, but the study population was smaller, and the performance was slightly lower^59^. In our study, the AUC values for chest-CT and RT-PCR increased from 0.778 to 0.892 and from 0.852 to 0.930 with the contribution of machine learning. The generalization of such models will allow increasing the diagnostic performances of both chest-CT and RT-PCR for COVID-19 diagnosis.

### Limitations

Our study has some limitations. First, the machine learning models developed in this experimentation are not directly transferrable to other hospitals due to it’s monocentric design. Such models must be further developed and tested on a larger scale to be generalized. However, the predictive variables selected and identified as highly important in this study are similar to the clinical and biological signs reported by previous authors, suggesting the absence of major obstacles for model generalization. Second, the study population included only hospitalized patients suspected to have COVID-19. It would be interesting to perform a similar study in non-hospitalized patient to test the model performances for COVID-19 diagnosis in paucisymptomatic patients. Finally, the classification of chest-CT as negative on the basis of the absence of typical images of COVID-19 might need to be reviewed in line with recent publications on COVID-19 diagnosis using deep learning methods.

### Conclusion

Our study demonstrates that machine learning models can be developed for improving COVID-19 diagnosis in patients hospitalized through the ED. Models based on chest-CT or RT-PCR will increase the performance of these tests by using clinico-biological variables. After generalization, machine learning should play a key role in the management of the outbreak by improving the performances of chest-CT and RT-PCR for COVID-19 diagnosis.

## Supplementary Information

Supplementary Information 1.

## Data Availability

After publication, the data will be made available to others on reasonable requests to the corresponding author.
