# Author Correction: Machine learning is the key to diagnose COVID-19: a proof-of-concept study

**DOI:** 10.1038/s41598-021-97049-1

**Published:** 2021-08-27

**Authors:** Cedric Gangloff, Sonia Rafi, Guillaume Bouzillé, Louis Soulat, Marc Cuggia

**Affiliations:** 1 Univ Rennes, CHU Rennes, INSERM, LTSI-UMR 1099, 35000 Rennes, France; 2 Department of Emergency Medicine, CHU Rennes, 35000 Rennes, France

Correction to: *Scientific Reports* 10.1038/s41598-021-86735-9, published online 30 March 2021

The original version of this Article contained errors in the Affiliations.

Affiliation 1 was incorrectly given as “LTSI Laboratory, INSERM U1099, Université de Rennes 1, Rennes, France”. The correct affiliation is listed below:

Univ Rennes, CHU Rennes, INSERM, LTSI-UMR 1099, F-35000 Rennes, France

Affiliation 2 was incorrectly given as “Department of Emergency Medicine, Pontchaillou University Hospital, 35033, Rennes, France”. The correct affiliation is listed below:

Department of Emergency Medicine, CHU Rennes, F-35000 Rennes, France.

The original Article and accompanying Supplementary Information file have been corrected

