# Supplementary material for: Machine learning is the key to diagnose COVID-19: a proof-of-concept study
Source: Sci Rep. 2021 Mar 30;11:7166. doi: 10.1038/s41598-021-86735-9 (PMC8009887; doi:10.1038/s41598-021-86735-9)
Supplement: Supplementary file 1 — Supplementary Information 1. [file 41598_2021_86735_MOESM1_ESM.docx]

**Machine learning is the key to diagnose COVID-19: a proof-of-concept study.**

**Cedric Gangloff, MD-PhD^1*^; Sonia Rafi, MD^1^ ; Guillaume Bouzillé, MD-PhD^1^ ; Louis Soulat, MD^2^ ; Marc Cuggia, MD-PhD^1^**

*Corresponding author: [cedric.ganglof@gmail.com](mailto:cedric.ganglof@gmail.com)

1: Univ Rennes, CHU Rennes, INSERM, LTSI-UMR 1099, F-35000 Rennes, France.

2: Department of Emergency Medicine, CHU Rennes, F-35000 Rennes, France.

**Supplementary table 1. Variables not considered as variables of interest.**

|  | NOT-COVID (n=430) | COVID (n=106) | p |
| --- | --- | --- | --- |
| Dyspnea, % | 72.78 ( 68.11 - 77.45 ) | 77.22 ( 67.97 - 86.46 ) | 0.5052 |
| Anosmia, % | 12.89 ( 9.38 - 16.41 ) | 13.92 ( 6.29 - 21.56 ) | 0.9518 |
| Falls, % | 18.91 ( 14.8 - 23.02 ) | 13.92 ( 6.29 - 21.56 ) | 0.3789 |
| Age, year | 68.6 ( 66.5 - 70.7 ) | 65.8 ( 62 - 69.6 ) | 0.7697 |
| NT-proBNP, pg/mL | 4279 ( 3181 - 5376 ) | 7234.1 ( 871 - 13596 ) | 0.3733 |
| Total bilirubin, μmol/L | 23.2 ( 15.4 - 31 ) | 24.8 ( 7.5 - 42.1 ) | 0.8434 |
| Calcemia, mmol/L | 2.2 ( 2.2 - 2.2 ) | 2.2 ( 2.1 - 2.3 ) | 0 |
| pH | 7.4 ( 7.4 - 7.4 ) | 7.4 ( 7.4 - 7.4 ) | 0.0668 |
| Partial Pressure of Oxygenn mHg | 97 ( 91.2 - 102.8 ) | 83.2 ( 74.1 - 92.3 ) | 0.2651 |
| Partial pressure of carbon dioxide, mmHg | 38.1 ( 36.9 - 39.3 ) | 35.8 ( 33.4 - 38.2 ) | 0.7818 |
| Oxyhemoglobin, % | 93.4 ( 92.5 - 94.3 ) | 91.9 ( 88.2 - 95.6 ) | 0.905 |
| Bicarbonate | 26.8 ( 26.2 - 27.4 ) | 25.7 mmol/L ( 24.5 - 26.9 ) | 0.9394 |
| Mean corpuscular hemoglobin concentration, g/dL | 33.6 ( 33.5 - 33.7 ) | 34 ( 33.7 - 34.3 ) | 0.3753 |
| Protidemia, g/L | 70.1 ( 68.9 - 71.3 ) | 68.6 ( 66.1 - 71.1 ) | 0.4752 |
| Gamma-glutamyl transferase, UI/L | 109.6 ( 80.3 - 138.9 ) | 93.3 ( 37.9 - 148.7 ) | 0.5729 |
| Alkaline phosphatase, IU/L | 101.3 ( 92.2 - 110.4 ) | 101.1 ( 84 - 118.2 ) | 0.8851 |
| C-reactive protein mg/L | 52.5 ( 43.1 - 61.9 ) | 51.9 ( 36.3 - 67.5 ) | 0.8574 |
| Aspartate aminotransferase, U/L | 64.5 ( 47.1 - 81.9 ) | 46.2 ( 33.9 - 58.5 ) | 0.1845 |
| Glycemia, mmol/L | 6.8 ( 6.4 - 7.2 ) | 6.3 ( 5.7 - 6.9 ) | 0.3259 |
| Urea, mmol/L | 7.7 ( 7 - 8.4 ) | 7.7 ( 6 - 9.4 ) | 0.449 |
| Creatinine, μmol/L | 93.9 ( 84.5 - 103.3 ) | 105.7 ( 85.9 - 125.5 ) | 0.489 |
| Mean corpuscular volume, fL | 91.4 ( 90.6 - 92.2 ) | 88.5 ( 86.6 - 90.4 ) | 0.5676 |
| Natremia, mmol/L | 138 ( 137.5 - 138.5 ) | 136.8 ( 135.6 - 138 ) | 0.7278 |
| Amount of hemoglobin per red blood cell, pg/cell | 30.7 ( 30.4 - 31 ) | 29.8 ( 29.1 - 30.5 ) | 0.7824 |
| Chloremia, mmol/L | 100.5 ( 99.9 - 101.1 ) | 100.1 ( 98.8 - 101.4 ) | 0.7878 |
| Red blood cell distribution width, % | 15.2 ( 14.9 - 15.5 ) | 14.8 ( 14.3 - 15.3 ) | 0.9053 |

Means and percentage between groups were compared with Student’s t- and chi-square tests, respectively. Variables with p >0.02 were not considered as variables of interest and represented in this table. Values in parentheses represent 95% confidence interval. *=p < 0.005.
